# Comparison of machine learning and deep learning techniques for the prediction of air pollution: a case study from China

**DOI:** 10.1007/s44273-023-00005-w

**Published:** 2023-05-26

**Authors:** Ishan Ayus, Narayanan Natarajan, Deepak Gupta

**Affiliations:** 1 Department of Computer Science and Engineering, ITER, Siksha ‘O’ Anusandhan University, Bhubaneswar, Odisha India; 2 Department of Civil Engineering, Dr. Mahalingam College of Engineering and Technology, Tamil Nadu, Pollachi, 642003 India; 3 Department of Computer Science & Engineering, MNNIT Allahabad, Prayagraj, 211004 India

**Keywords:** AQI, Bidirectional GRU, Bidirectional LSTM, CNN BiLSTM, Conv1D BiLSTM

## Abstract

The adverse effect of air pollution has always been a problem for human health. The presence of a high level of air pollutants can cause severe illnesses such as emphysema, chronic obstructive pulmonary disease (COPD), or asthma. Air quality prediction helps us to undertake practical action plans for controlling air pollution. The Air Quality Index (AQI) reflects the degree of concentration of pollutants in a locality. The average AQI was calculated for the various cities in China to understand the annual trends. Furthermore, the air quality index has been predicted for ten major cities across China using five different deep learning techniques, namely, Recurrent Neural Network (RNN), Bidirectional Gated Recurrent unit (Bi-GRU), Bidirectional Long Short-Term Memory (BiLSTM), Convolutional Neural Network BiLSTM (CNN-BiLSTM), and Convolutional BiLSTM (Conv1D-BiLSTM). The performance of these models has been compared with a machine learning model, eXtreme Gradient Boosting (XGBoost) to discover the most efficient deep learning model. The results suggest that the machine learning model, XGBoost, outperforms the deep learning models. While Conv1D-BiLSTM and CNN-BiLSTM perform well among the deep learning models in the estimation of the air quality index (AQI), RNN and Bi-GRU are the least performing ones. Thus, both XGBoost and neural network models are capable of capturing the non-linearity present in the dataset with reliable accuracy.

## Introduction

Air pollution has become a significant concern in several countries. The issue of urban air pollution has grown more acute as a result of the fast urbanization and acceleration of industrialization. It has adversely affected our physical health and living environment. This is caused due to excessive release of dangerous substances into the atmosphere, such as greenhouse gases, particulates, and biological compounds (Goudarzi et al., 2019; Khaefi et al., 2016). The presence of pollutants in the air can cause allergies, illnesses, and even death (He et al., 2019; Liu et al., 2018). Urbanization and industrialization have tremendously augmented air pollution (Lin & Zhu, 2018). The public is sensitive even to marginal changes in air quality as it has a severe impact on human health (Khaniabadi et al., 2017; Zou et al., 2019). Air pollution control is complex with no rigid environmental regulations (Kumar et al., 2022).

As a result, research on forecasting air quality is crucial and has always been seen as a crucial issue in environmental preservation. It serves as a key tool for guiding scientific judgement in air pollution warning and management decisions. To track a city’s PM_2.5_ and other air pollutants in real time, several large cities have installed air quality monitoring stations. A major challenge of the work of forecasting air quality is the early diagnosis of air pollution incidence and PM_2.5_ concentration value progression.

Several studies have been conducted for the prediction of individual air quality parameters (Wu et al., 2011; Li et al. 2016; Li et al., 2017; Wen et al., 2019; Al-Janabi et al. 2020; Gu et al. 2020), which are measured in the units of parts per million or micrograms per cubic metres. In comparison with the individual parameters, the Air quality index (AQI) is a comprehensive index that measures air quality in a quantitative manner, obtained by integrating numerous air quality pollutants. This is estimated with reference to the new ambient air quality standards GB2095-2012 for China (Zhu et al., 2017). The AQI is a unique indicator that enables us to closely monitor the influence of air quality on health (Ribeiro et al., 2016). The lower value of AQI guarantees better human health, and vice versa. Table 1 provides the air quality index classification for China. Real-time AQI facilitates air pollution control and the protection of human health (Ni et al., 2017). Early prediction of AQI plays a vital role in the decision-making for environmental management and prevention of upcoming dangers due to air pollution (Jiang et al., 2018; Pisoni et al., 2018).

**Table 1:** AQI classification for China (Gao, 2013)

AQI index	AQI air quality status	Air quality grade
0–50	Excellent	First level
51–100	Good	Second level
101–150	Light pollution	Third level
151–200	Moderate pollution	Fourth level
201–250	Heavy pollution	Fifth level
251–300	Serious pollution	Sixth level

In the past, approaches such as Artificial Neural Networks (ANN) (Song et al., 2015; Patra et al., 2016; Perez et al. 2016; Biancofiore et al., 2017), Support Vector Machine (SVM) (Osowski & Garanty, 2007), and Adaptive Neuro-Fuzzy Inference System (ANFIS) (Taylan 2017) were used to predict the air quality parameters. Recently, Shahriar et al. (2020) used machine learning models such as Linear Support Vector Machine (L-SVM), ANN, Gaussian Process Regression (GPR), Medium Gaussian-Support Vector Machine (M-SVM), time series model PROPHET, and Random Forest Regression (RFR), for the prediction of oxides of nitrogen (NO_X_), ozone (O_3_), sulphur dioxide (SO_2_), and carbon monoxide (CO) along with meteorological parameters from Dhaka, Rajshahi, Chattogram, and Sylhet. Later, Shaziayani et al. (2021) determined the best loss function between Quantile Regression (QR) and Ordinary Least Squares (OLS) using Boosted Regression Tree (BRT) for the prediction of particulate matter with 10 microns (PM_10_) concentration in Malaysia. Jing et al. (2020) have utilized the XGBoost model of machine learning to study the hourly prediction of AQI for Shijiazhuang, Hebei province. Kumar et al. (2022) have investigated the performance of various machine learning models for the analysis of 6 years of air pollution data across 23 stations in India where XGBoost performs best among the considered models.

While both machine learning and deep learning use data for feature learning, deep learning differs considerably from machine learning due to its ability to scale with data. Machine learning systems generally experience a performance plateau after training on large data sets before diminishing returns occur. However, as the size of the training datasets increases, deep learning models perform better. Deep learning automatically performs feature extraction and modelling following data training, whereas machine learning requires data scientists or users to do it. Traditional machine learning models cannot solve some problems, but deep learning models can.

In recent decades, deep learning (DL) has picked up momentum since it considers a multi-level learning process, where learning occurs at each level for a particular part of the problem, and the conglomeration of the results enables to solve the overall problem (Al-Janabi et al. 2020). In Ong et al. (2015), proposed a Deep Recurrent Neural Network (DRNN) enhanced with a novel pre-training technique, auto-encoder for the prediction of PM_2.5_ concentrations in Japan. In 2006, Li et al. proposed a novel Spatio-Temporal Deep Learning (STDL)-based air quality prediction method that uses a stacked autoencoder to extract inherent air quality features to predict PM_2.5_. They reported that their proposed method performed better compared to Spatio-Temporal ANN (STANN), Spatio-Temporal Support Vector Regression (STSVR), and Spatio-Temporal Autoregressive Moving Average (STARMA). Freeman et al. (2018) predicted 8-h averaged surface ozone (O_3_) concentrations using deep learning consisting of a recurrent neural network (RNN) with long short-term memory (LSTM). They found that the LSTM was able to forecast the duration of continuous O_3_ exceedances as well. In Jiao et al. (2019), predicted the AQI using Long Short-Term Memory (LSTM) with the help of PM_2.5_, PM_10_, sulphur dioxide (SO_2_), wind direction, nitrogen dioxide (NO_2_), carbon monoxide (CO), and ozone (O_3_). They concluded that LSTM is capable of the prediction of AQI. In 2020, Al-Janabi et al. proposed a new predictor based on LSTM and Particle Swarm Optimisation (PSO) to predict the concentration of six types of air pollutants. Dhakal et al. (2021) used deep LSTM to predict PM_2.5_ in Kathmandu Valley, Bangladesh, accurately. Many researchers have also analysed various recurrent deep neural networks such as RNN, GRU, and LSTM networks for identifying efficient predictive models (Athira et al., 2018; Navares & Aznarte, 2020).

Literature review reveals that various deep learning techniques have been employed to predict the air quality parameters but the performance comparison of various recurrent deep learning, hybrid deep learning, and machine learning techniques is yet to be explored. From past studies, it is quite evident that machine learning and deep learning techniques are widely used for various studies. In certain circumstances, machine learning performs well. While in other circumstances, deep learning models present their best performance. A hybrid model is created based on the combination of their better halves. The hybrid deep learning techniques are found to be useful for modelling the uncertainty by the fusion of deep neural networks along with a probabilistic approach. These models are more advantageous than any other model because they maintain the dual effect of the original model. In this study, we have compared various recurrent-based networks to identify a better predictive model for predicting AQI.

The objective of the study is to compare various machine and deep learning techniques for predicting the air quality index. For this purpose, we have considered the data from various stations in China. The researchers have suggested that among the various machine learning models the XGBoost is considered the most suitable machine learning model for prediction and hence, considered in this study. Similarly, among the recurrent deep neural network-based models, simple recurrent neural networks, bidirectional gated recurrent networks, bidirectional long short-term memory networks, etc., have been considered. All the above-mentioned models have been compared with hybrid deep learning models such as Convolutional LSTM and Convolutional neural network- LSTM. In the present study, we have analysed the five different deep learning (both recurrent and hybrid) techniques, namely, Bidirectional-GRU (Bi-GRU), Bidirectional LSTM (Bi-LSTM), Recurrent Neural Network (RNN), CNN-BiLSTM, and Convolutional BiLSTM (Conv1D-BiLSTM), along with a machine learning technique, i.e. XGBoost for comparison of their accuracy in forecasting AQI. This study performs a detailed analysis on the performance of the above-mentioned models for ten stations across China. In essence, the behaviour of various predictive models has been compared for the efficient prediction of AQI in China.

### Study area and description

**Fig. 1:**
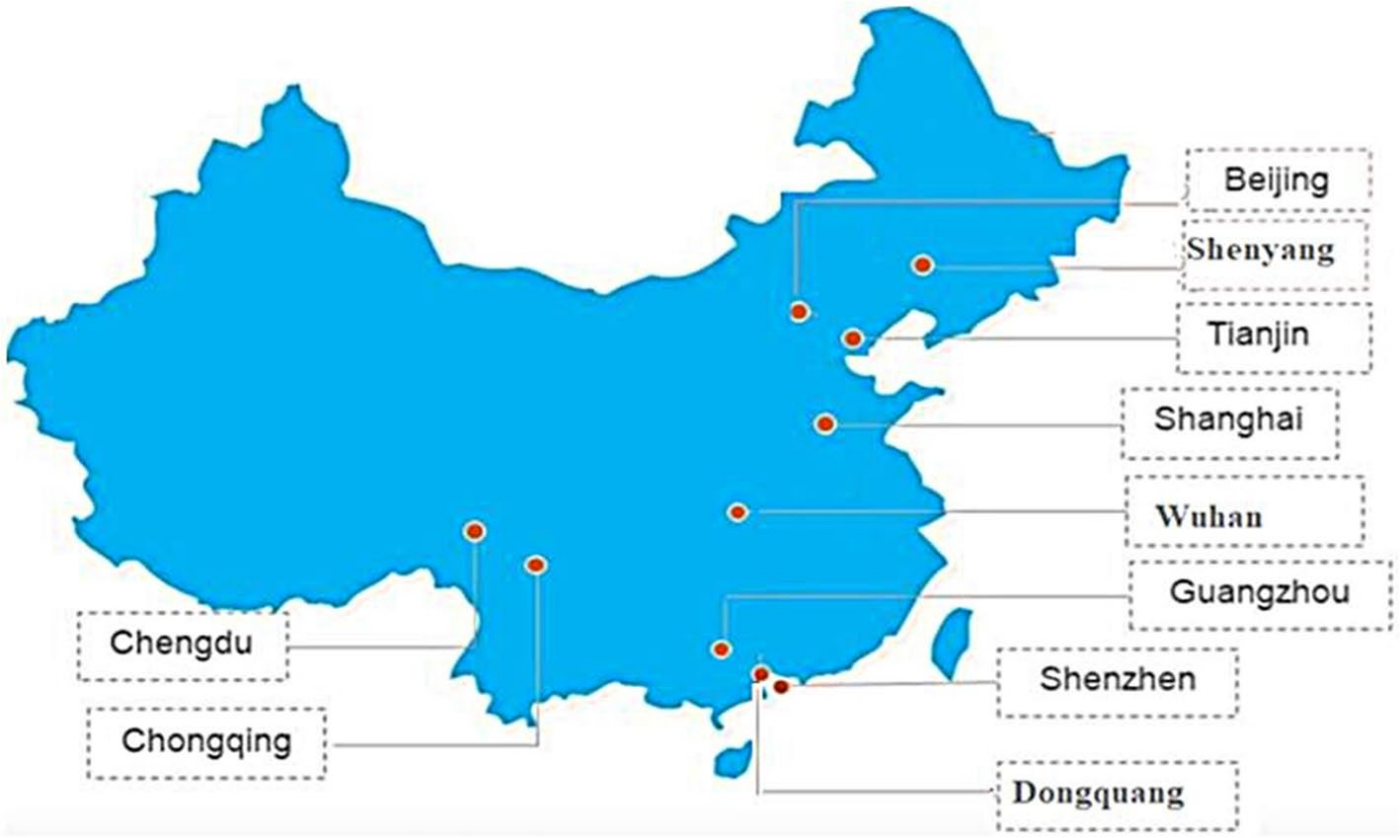
Geographical map of China with the chosen stations (in boxes)

China is located in the Southeast region of Asia (Agency, 2011) with the world’s highest population. To meet the increasing needs of the growing population, China had to increase investments in various developmental projects. The surge in various goods manufacturing factories and the increasing number of motorized vehicles are potential factors for rising air pollution. The alarming situation caused due to the increasing air pollution needs to be handled carefully for the betterment of people. According to an earlier study, China possesses a higher distribution of population density in the southeast region when compared to the northwest (Minmin, et al. 2018). Therefore in this study, we have considered ten major stations in the most populated regions of China. These cities include Beijing, Chengdu, Chongqing, Dongguan, Guangzhou, Shanghai, Shenyang, Shenzhen, Tianjin, and Wuhan. The location of the various stations is shown in Fig. 1. The geographical details such as latitude, longitude, and elevation of the selected stations are provided in Table 2.

**Table 2:** Geographical details of the selected stations

Sl. No	Name of the city	Latitude (°N)	Longitude (°E)	Elevation (m)
1	Beijing	39.90	116.40	44
2	Chengdu	30.57	104.06	500
3	Chongqing	29.43	106.91	244
4	Dongguan	23.02	113.75	8
5	Guangzhou	23.12	113.26	21
6	Shanghai	31.23	121.47	4
7	Shenyang	41.80	123.43	55
8	Shenzhen	22.54	114.05	1
9	Tianjin	39.34	117.36	5
10	Wuhan	30.59	114.30	37

It is observed from Table 2 that most of the stations are lying in very low altitudes (Shenzhen, Shanghai, Tianjin, and Dongguan), while some of them are situated higher (Shenyang, Wuhan, Guangzhou, and Beijing). Among the chosen stations, Chongqing and Chengdu are situated at higher elevations of 244 m and 500 m. Hence, the current study takes into account stations located at various altitudes.

### Methodology

**Fig. 2:**
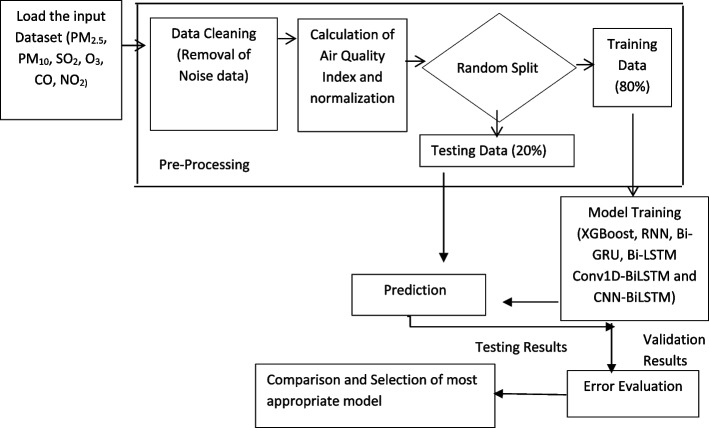
Schematic representation of the proposed methodology for the prediction of AQI

This study evaluates six different models using a variety of deep learning and machine learning methods, including CNN-BiLSTM, Conv1D-BiLSTM, RNN, BiLSTM, Bi-GRU, and XGBoost. We have compared and identified the most appropriate model for efficient forecasting of air quality. Figure 2 presents a schematic flow diagram representing the methodology for air quality prediction. The methodology in this study includes collecting and loading the air quality datasets, data pre-processing, model building, model training, prediction, performance evaluation, and selection of the most suitable model. Initially, the air quality data was collected for analysis. The study involved a series of data preprocessing steps aimed at cleaning noisy data, followed by the calculation of the Air Quality Index (AQI) and the division of the dataset into training and testing sets. The preprocessed data contained information on PM_2.5_, carbon monoxide (CO), sulphur dioxide (SO_2_), PM_10_, nitrogen dioxide (NO_2_), and ozone (O_3_), along with their corresponding AQI values, for a duration of 24 h. Machine learning models such as XGBoost, and deep learning models such as RNN, BiLSTM, and BiGRU, as well as hybrid models such as CNN-BiLSTM and Conv1D-BiLSTM, were employed for training. These models were trained successfully on the preprocessed data. During the testing phase, models were evaluated using pollutant data from the testing set to determine their predictive accuracy of AQI. The predicted and observed data were then compared to produce performance metrics, and the performance of each predictive model was compared to identify the most appropriate model for accurate AQI prediction.

#### Air quality dataset

In this study, we examined daily air quality data obtained from ten different cities in China, namely, Beijing, Shanghai, Chongqing, Dongguan, Shenyang, Shenzhen, Chengdu, Guangzhou, Tianjin, and Wuhan. The data spanned more than 7 years, specifically, from December 31, 2013, to May 6, 2021. The data was collected from the website https://aqicn.org comprising several crucial variables, namely, PM2.5 (particle diameter ≤ 2.5 µm), PM10 (particle diameter ≤ 10 µm), sulphur dioxide (SO2), nitrogen dioxide (NO2), ozone (O3), and carbon monoxide (CO), which was deemed necessary for accurately predicting the air quality index (AQI).

#### Data pre-processing

Data pre-processing is one of the essential stages for any data analysis problem where the noise present in the datasets is detected and removed. The data pre-processing consists of three different sub-steps, i.e. data cleaning, AQI calculation, and splitting of the dataset into training and testing sets. Initially, the process of data cleaning was performed on the raw data, where the missing values present in the dataset are replaced. We have considered a simple imputation technique using a central tendency measure for filling in the missing value. When outliers are present in the observed data, using the median is more reliable than the mean. Hence, the median values are considered to replace the missing values for various pollutant parameters such as PM_2.5_, PM_10_, SO_2_, O_3_, CO, and NO_2_ data. The duplicate observations and outliers were also identified and removed. Further, the AQI values were calculated using various air pollutant parameters for selected stations in China.

The AQI value is an essential index value used for determining the quality of air in a particular region. The calculated AQI was added to the dataset as a target variable. Data are normalized to deal with variable significance and improve the model learning rate. To normalize the data, in this study, we have used min–max normalization which performs a linear transformation on the given data. Multiple variables are normalized by normalizing each variable (PM_2.5_ (particle diameter ≤ 2.5 µm), PM_10_ (particle diameter ≤ 10 µm), sulphur dioxide (SO_2_), ozone (O_3_), nitrogen dioxide (NO_2_), and carbon monoxide (CO)) separately. Normalization retains the pattern of data by scaling down the data to the range of 0–1. The relationship between the various variables is preserved in normalization. Mathematically,

1 $$\begin{array}{ll}{U}_{X}^{^{\prime\prime} }=\frac{{U}_{X}-{\mathrm{MIN}}_{K}}{{\mathrm{MAX}}_{K}-{\mathrm{MIN}}_{K}}\left({\mathrm{MAX}}_{K}^{^{\prime\prime} }-{\mathrm{MIN}}_{K}^{^{\prime\prime} }\right)+{\mathrm{MIN}}_{K}^{^{\prime\prime} }&,\mathrm{ where }{\mathrm{MAX}}_{K}^{^{\prime\prime} }=1\ and\ {\mathrm{MIN}}_{K}^{^{\prime\prime} }=0\\{U}_{X}^{^{\prime\prime} }=\frac{{U}_{X}-{\mathrm{MIN}}_{K}}{{\mathrm{MAX}}_{K}-{\mathrm{MIN}}_{K}}&\end{array}$$

Here ${MAX}_{K}$ represents the maximum value of a variable *K*, ${MIN}_{K}$ represents the minimum value of a variable *K*, ${U}_{X}$ is the input value, ${MAX}_{K}^{^{\prime\prime} }$ is the new maximum value which is equal to 1, and ${MIN}_{K}^{^{\prime\prime} }$ is the new minimum value which is equal to 0 (Han et al., 2012). Again ${U}_{X}^{^{\prime\prime} }$ presents the normalized values of each data entry on the basis of respective variables. The considered variables are essential for better forecasting of the air quality index. Therefore, these variables normalized each entry of the variable by utilizing the minimum (${\mathrm{MIN}}_{K})$ and maximum (${\mathrm{MAX}}_{K}$) values of the respective variable. Again here *K* ranges from 0 to 5 where *K* = 0 refers to PM_2.5_, K = 1 refers to PM_10_, *K* = 2 refers to SO_2_, *K* = 3 refers to O_3_,* K* = 4 refers to NO_2_, *K* = 5 refers to CO. Further, the normalized dataset is ready for random splitting where 80% of the total dataset is considered as training dataset and rest 20% of the total dataset is considered as testing dataset.

#### AQI calculation

The Air Quality Index (AQI) measures the status of air quality. The index value of AQI generally ranges from 0 to 500, where the highest index value indicates the presence of harmful air pollutants in the air, thus causing adverse health effects on the common people. Similarly, the presence of the lowest index value ensures the presence of the best quality of air in the atmosphere. The input data is composed of the various air pollutant concentrations as a variable such as SO_2_, CO, NO_2_, O_3,_ PM_2.5_, and PM_10_. These input variables have various constraints for different air pollutants such as O_3_ which is collected with a maximum of 8 h while SO_2_, CO, NO_2_, PM_2.5_, and PM_10_ are collected with an average concentration of 24 h as shown in Table 3. The computation of AQI (average of 24 h) is a two-step process as given below.

(i) Individual -index for each pollutant. Mathematically, the sub-index (${I}_{s-i}$) is given by
  2 $${I}_{s-i}=\left\{\frac{{I}_{\mathrm{High}}-{I}_{\mathrm{Low}}}{{B}_{\mathrm{High}}-{B}_{\mathrm{Low}}}\right\}*\left({P}_{c}-{B}_{\mathrm{Low}}\right)+{I}_{\mathrm{Low}}$$

where ${P}_{c}$ is the pollutant concentration, ${B}_{\mathrm{High}}$ is the breakpoint concentration which is either greater than or equal to the given concentration ${P}_{c}$, ${B}_{\mathrm{Low}}$ is the breakpoint concentration which is either smaller or equal to the given concentration ${P}_{c}$, ${I}_{\mathrm{High}}$ is the breakpoint index for ${B}_{\mathrm{High}}$, ${I}_{\mathrm{Low}}$ is the breakpoint index for ${B}_{\mathrm{Low}}$. The calculated sub-index of each pollutant concentration describes their influence on human health. And the calculated sub-index of each pollutant is utilized to determine an overall AQI value of the basis of different pollutants such as SO_2_, CO, NO_2_, O_3,_ PM_2.5_, and PM_10._

**Table 3:** Breakpoint concentration (Gao, 2013)

AQI category (range)	PM _2.5_ (24 h)	PM_10_ (24 h)	O_3_ (8 h)	NO_2_ (24 h)	SO_2_ (24 h)	CO (24 h)
Excellent (0–50)	0–35	0–50	0–100	0–40	0–50	0–2
Good (51–100)	36–75	51–150	101–160	41–80	51–150	3–4
Lightly polluted (101–150)	74–115	151–250	161–215	81–180	151–475	5–14
Moderately polluted (151–200)	116–150	251–350	216–265	181–280	476–800	15–24
Heavily polluted (201–300)	151–250	351–420	266–800	281–565	801–1600	25–36
Severely polluted (301–500)	250 +	420 +	-	565 +	1601–2620	36 +

(ii) The sub-indices for pollutant concentration are aggregated to determine the overall AQI values by using weighted average method as given below.

3 $$\mathrm{AQI}=\mathrm{WA}\left\{{I}_{s-i}\left({\mathrm{PM}}_{2.5}\right), { I}_{s-i}\left({\mathrm{PM}}_{10}\right), { I}_{s-i}\left({\mathrm{O}}_{3}\right), { I}_{s-i}\left({\mathrm{SO}}_{2}\right), { I}_{s-i}\left(\mathrm{CO}\right), { I}_{s-i}\left({\mathrm{NO}}_{2}\right)\right\}$$

where ${I}_{s-i}$(PM_2.5_) is the sub-index value for PM_2.5_ with an average of 24-h concentration, ${I}_{s-i}$(PM_10_) is the sub-index value for PM_10_ with an average of 24-h concentration, ${I}_{s-i}\left({\mathrm{O}}_{3}\right)$ is the sub-index value for O_3_ with a maximum of 8-h concentration, ${I}_{s-i}\left({\mathrm{SO}}_{2}\right)$ is the sub-index value for SO_2_ with an average of 24-h concentration,${ I}_{s-i}\left(\mathrm{CO}\right)$ is the sub-index value for CO with an average of 24-h concentration, and ${I}_{s-i}\left({\mathrm{NO}}_{2}\right)$ is the sub-index value for NO_2_ with an average of 24-h concentration. And WA refers to the weighted average method to calculate AQI value. The AQI index was calculated as output data concentration for an average duration of 24 h.

#### Machine learning and deep learning models

##### XGBoost

Extreme Gradient Boosting is an efficient Gradient Boosting Decision Tree (GBDT) algorithm with the perfect combination of hardware and software optimization techniques that produce better results with efficient usage of memory. This model prevents the condition of overfitting as it supports both LASSO (L1) and Ridge (L2) regularization. The use of regularization, sparsity awareness, weighted quantile sketch algorithm, and cross-validation results in algorithmic enhancement. The system optimization is assisted by parallelization, tree pruning, and hardware optimization (Chen & Guestrin, 2016). At each iteration, the XGBoost algorithm computes a set of features that are most important for predicting the target variable. These features are used to split the data into smaller subgroups based on their values. The subgroups are split until the model can no longer make improvements, or until a predefined stopping criterion is met. The goal of the XGBoost algorithm is to learn a function that can predict a continuous numerical value given a set of input features. To increase the model’s accuracy, the XGBoost method iteratively adds decision trees to it. The decision trees are built using a process called boosting, where each subsequent tree is built to correct the errors of the previous trees. The boosting process is done in a way that each new tree focuses on the samples that were misclassified by the previous trees. The tuning of parameters and other influential factors is responsible for winning an algorithm. The model has been implemented for a wide range of parameters to choose the best-performing parameter for the problem.

##### Recurrent neural network

A short-term memory network formed from a feed-forward neural network is referred to as RNN. The connection between the different nodes and backward loops enables the network to remember the present and the recent past. Thus, the model retains essential information of input data allowing precise prediction of the corresponding output. Mathematically, the output from the different layers is given by

4 $${H}_{t}={W}_{HI} {I}_{t}+ {W}_{HH}{H}_{t-1}+{b}_{H}$$

5 $${O}_{t}={W}_{HO}{H}_{t}+{b}_{O}$$

where $I =\left\{{I}_{1}+{I}_{2}+{I}_{3}+\dots +{I}_{t}\right\}$ represent input layer sequence, $H=\left\{{H}_{1}+{H}_{2}+{H}_{3}+\dots +{H}_{t}\right\}$ represents hidden layer sequence, and $O=\left\{{O}_{1}+{O}_{2}+{O}_{3}+\dots +{O}_{t}\right\}$ represents output layer sequence.${W}_{HO}$ is the hidden-output weights,${W}_{HH}$ is the hidden-hidden weight,${W}_{HI}$ is the input-hidden weight, ${I}_{t}$ is the current input data,${H}_{t}$ is the new state of the hidden layer, ${H}_{t-1}$ is the previous state of the corresponding hidden layer,${b}_{H}$ is bias at the hidden layer, and ${b}_{O}is$ bias at the output layer. The RNN models do suffer from the problem of vanishing gradient, i.e. as the parameters are updated the gradient becomes smaller and smaller, and gradually the parameters become insignificant affecting the learning rate of the long data sequence (Nejadettehad et al., 2020). In this study, we have used 2 simple RNN layers followed by a dense layer. The parameters like Adam optimizer, number of epochs as 200, learning rate as 1e − 8, and decay as 1e − 9 are utilized by the designed model.

##### Gated recurrent units

GRUs is a special type of recurrent neural network that has two primary gates, the update gate and the reset gate. The memory of the network is controlled using these gates. The update gate permits us to control the amount of the new state as just the copy of the old state whereas the reset gate permits us to control the amount of the previous state we want the network to remember. Mathematically, if ${I}_{t}$ represents the input state of given time step and ${H}_{t-1}$ represents the hidden state of previous time step, then output at reset gate, i.e.${RG}_{t}$; output at update gate, i.e.${UG}_{t}$; candidate hidden state, i.e.$\widetilde{{CH}_{t}}$; and final update equation, i.e.${H}_{t}$, are given by

6 $${RG}_{t}=\sigma ({I}_{t}{W}_{IR}+{H}_{t-1}{W}_{HR}+{b}_{R})$$

7 $${UG}_{t}=\sigma ({I}_{t}{W}_{IU}+{H}_{t-1}{W}_{HU}+{b}_{U})$$

8 $${\widetilde{CH}}_{t}=\mathrm{tanh}\left[{I}_{t}{W}_{IC}+\left({RG}_{t}\odot {H}_{t-1}\right){W}_{HC}+{b}_{C}\right]$$

9 $${H}_{t}={UG}_{t}\odot {H}_{t-1}+(1-{UG}_{t})\odot {CH}_{t}$$

where ${b}_{R}$, ${b}_{U},{b}_{C}$ are biases and ${W}_{IR}$, ${W}_{HR}$, ${{W}_{IU}, W}_{HU},$ ${W}_{IC}, {W}_{HC}$ are weight parameters. The Hadamard product operator is represented by the symbol ⨀. The sigmoid function is used to remodel the input data to the interval (0–1) (Zhou et al., 2019a, 2019b). The following parameters are considered for this model, Adam optimizer, number of epochs as 200, learning rate as 1e − 3, and decay rate as 1e − 9.

##### Bidirectional gated recurrent units

Bi-GRU is a bidirectional structure with the ability to learn the data sequence from both directions. This model is a combination of two unidirectional GRUs. One of these GRU models allows learning in a forward direction, i.e. from the beginning of the data sequence whereas the other GRU allows learning in a backward direction, i.e. from the end of the data sequence. Mathematically, the definition of Bi-GRU can be expressed as:

10 $$\overrightarrow{{H}_{t}}={GRU}_{forward}({I}_{t},\overrightarrow{{H}_{t-1}})$$

11 $$\overleftarrow{{H}_{t}}={GRU}_{backward}({I}_{t},\overleftarrow{{H}_{t+1}})$$

12 $${H}_{t}= \overrightarrow{{H}_{t}} \otimes \overleftarrow{{H}_{t}}$$

where $\overrightarrow{{H}_{t}}$ represents the final state of forward GRU, $\overleftarrow{{H}_{t}}$ represents the final state of backward GRU, and ⨂ symbol represents the concatenation operation (Zhou et al., 2019a, 2019b). In this study, we have used two layers of Bi-GRU followed by dense layers. The following parameters are considered for this model, Adam optimizer, number of epochs as 200, learning rate as 1e − 3, and decay rate as 1e − 9.

##### Long short-term memory network

A variant of RNN with the ability to solve long-term dependency problems is referred to as LSTM. LSTM comprises different gates like forget, input, and output gate which retains or remove information from the given cell state. The different mathematical operations used in each layer are as follows

13 $${FG}_{t}=\sigma ({W}_{x}[{H}_{t-1},{I}_{t}]{+b}_{x})$$

14 $${IG}_{t}=\sigma ({W}_{x}[{H}_{t-1},{I}_{t}]{+b}_{x})$$

15 $${CS}_{t}= {f}_{t}*{CS}_{t-1}+{IG}_{t}*\mathrm{tanh}({W}_{x}[{H}_{t-1},{I}_{t}]{+b}_{c})$$

16 $${OG}_{t}=\sigma ({W}_{x}[{H}_{t-1},{I}_{t}]{+b}_{x})$$

17 $${H}_{t}={OG}_{t}*\mathrm{tanh}({CS}_{t})$$

where ${W}_{x}$ is the weight, ${b}_{x}\& {b}_{c}$ are the biases, ${I}_{t}$ is the input, ${H}_{t-1}$ is the output of previous state, ${FG}_{t}$ is the output of forget gate, ${IG}_{t}$ is the output of input gate, ${OG}_{t}$ is the output of output gate, and ${CS}_{t}$ is the cell state (Xayasouk et al., 2020). The different variants of LSTM networks considered in this study are BiLSTM, CNN-BiLSTM, and Conv1D-BiLSTM. These models have some common parameters such as number of epochs as 200, learning rate as 1e − 8, and decay as 1e − 9. The Adam optimizer was found to be the most suitable type of optimizer for the given problem.

##### Bidirectional long short-term memory network

The bi-LSTM model is a unique LSTM model with the ability to capture both forward and backward information from input data. This network runs the input sequence in two ways, i.e. from past to future as well as from future to past. The corresponding outputs are concatenated before passing to the next layer. The bidirectional network learns both from past and future data to predict the current state precisely (Li et al., 2018a, 2018b; Zhang et al., 2021). A bi-LSTM network is designed using two layers of bidirectional LSTM layer with the default merge mode, i.e. ‘concat’ followed by dense layers.

18 $${{H}_{t} =\mathrm{LSTM}}_{\mathrm{forward}}\left({I}_{t},\overrightarrow{{H}_{t-1}}\right) \otimes {\mathrm{LSTM}}_{\mathrm{backward}}({I}_{t},\overleftarrow{{H}_{t+1}})$$

where ⨂ symbol represents the concatenation operation, ${I}_{t}$ is the input, ${H}_{t-1}$ is the output of past state, and ${H}_{t+1}$ is the output of future state.

##### CNN-BiLSTM

CNN-BiLSTM is a hybrid model which uses a convolutional neural network for feature extraction and the bidirectional LSTM model to perform sequence prediction. This model is temporally and spatially deep and flexible enough to solve various problems of prediction. The convolution operation determines the relationship between two functions (Lu et al., 2020). The implementation of the CNN-BiLSTM model uses a 1D convolutional network which consists of Conv1D and MaxPooling1D layers, two BiLSTM layers followed by two dense layers. The Conv1D layer is assigned with the following parameters, namely, kernel size as 5, filters as 32, activation function as relu, and strides as 1. The Maxpooling1D layer uses a single parameter pool size which is assigned as 2.

##### Conv1D-BiLSTM

Conv1D-BiLSTM is a special kind of LSTM with an encoding–forecasting structure (Shi et al., 2015). The Conv1D-BiLSTM layer has been implemented with the Conv1D layer, two BiLSTM layers followed by two dense layers. This model performs padding just before the convolution operation to ensure that the state has the same number of rows and columns as that of input data. The different parameters used by the conv1D layer includes filters as 32, activation function as relu, kernel size as 5, and strides as 1.

#### Performance indicators

The reliability of different models is evaluated using different statistical indices such as Mean Square Error (MSE), Index of Agreement (IA), Mean Absolute Error (MAE), Root Mean Square Error (RMSE), and Symmetric Mean Absolute Percentage Error (SMAPE). They are mathematically defined as follows:

19 $$\mathrm{MAE}=\frac1K{\textstyle\sum_{i=1}^k}\vert Y_i^{real}-Y_i^{pred}\vert$$

20 $$\mathrm{MSE}=\frac1K{\textstyle\sum_{i=1}^k}({Y_i^{real}-Y_i^{pred})}^2$$

21 $$\mathrm{RMSE}=\sqrt{\frac1K{\textstyle\sum_{i=1}^k}({Y_i^{real}-Y_i^{pred})}^2}$$

22 $$IA=1-\frac{\sum_{i=1}^{k}{({Y}_{i}^{real} -{Y}_{i}^{pred})}^{2}}{\sum_{i=1}^{k}{(|{Y}_{i}^{real}-\overline{{Y}_{i}^{real}}|+|{Y}_{i}^{pred}-\overline{{Y}_{i}^{real}}|)}^{2}}$$

23 $$\mathrm{SMAPE}=\frac{100\%}K{\textstyle\sum_{i=1}^k}\frac{\vert Y_i^{pred}-Y_i^{real}\vert}{{(\vert Y}_i^{real}\left|+\left|Y_i^{pred}\right|\right)/2}$$

where *K* is the number of samples, ${Y}_{i}^{real}$ is the actual AQI level, $\overline{{Y}_{i}^{real}}$ is the average of original AQI level, ${Y}_{i}^{pred}$ is the forecasted AQI level, $\overline{{\mathrm{Y}}_{\mathrm{i}}^{pred}}$ is the average of forecast AQI level, and IA $\epsilon [\mathrm{0,1}]$. In this study, we compared a number of performance indicators for predicted and measured Air Quality Index (AQI) values for the time period from 17 November 2019 to 6 May 2021.

## Results and discussion

### Trend analysis

**Fig. 3:**
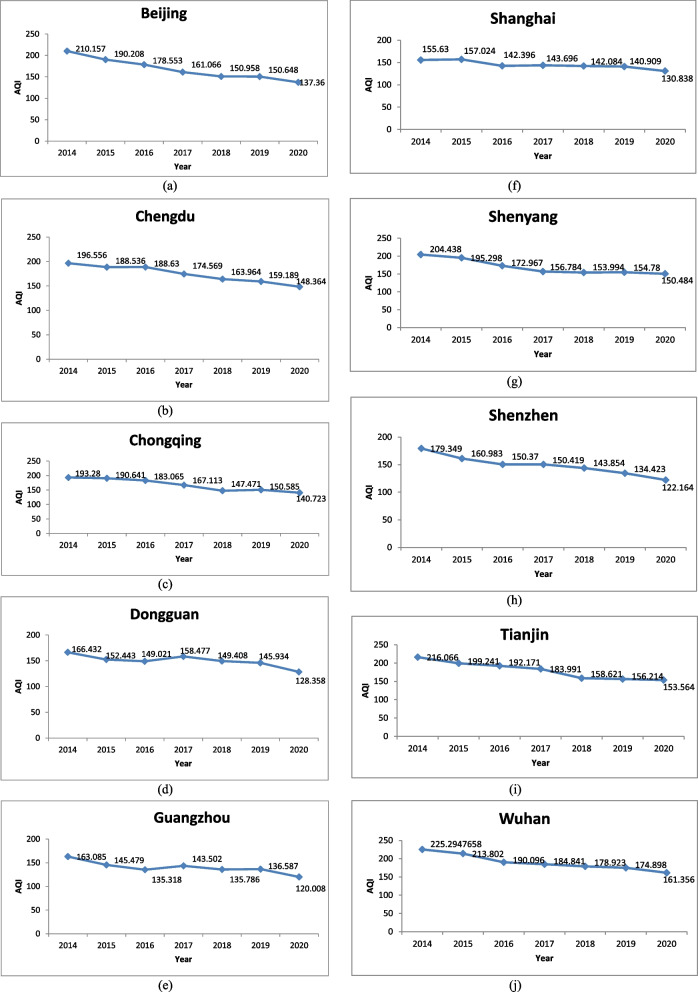
**a**–**j** AQI trend from 2014 to 2020 for the various cities in China

In this section, the average annual AQI trends are analysed for the selected stations, namely, Beijing, Chengdu, Chongqing, Dongguan, Guangzhou, Shanghai, Shenyang, Shenzhen, Tianjin, and Wuhan from the period of 2014 to 2020. From Fig. 3, it is observed that the annual AQI of all the selected cities has improved over the years except for a year or two in between. It is observed that most of the stations (Beijing, Chengdu, Chongqing, Dongguan, Guangzhou, Shenyang, Shenzhen, Tianjin, and Wuhan) are having a poor average air quality index in the year 2014. Shanghai is having the same in the year 2015. All the chosen stations have the best average air quality index in the year 2020 due to the imposition of lockdown to prevent the spread of the COVID-19 virus.

In this study, the calculation of the Air Quality Index (AQI) value was conducted during the pre-processing stage. Subsequently, the pollutant data (SO2, CO, NO2, O3, PM2.5, and PM10) was employed as feature variables to training the models, and the calculated AQI was considered as the target variable. The models were utilized to forecast the predicted AQI value, which was subsequently compared with the calculated AQI value utilizing the pollutant data. The actual predicted values and measured values of the parameters in this scenario depend on the specific considered models and dataset being used. The measured values of these pollutants would be the actual values measured by sensors located throughout the city, expressed in units such as μg/m3 or parts per million (ppm). The calculation of the Air Quality Index (AQI) involves a two-step process as described by Eqs. 2 and 3. In the first step, sub-indices are computed for each individual pollutant. Subsequently, the sub-indices are aggregated (weighted average method) and used to determine the AQI for a 24-h period. The actual predicted values, on the other hand, would be the predicted AQI values for a period of 24-h predicted based on the air pollutant measurements. To make these predictions, various machine learning models and deep learning models are considered taking into account the air pollutant measurements as input features and output predicted AQI values.

The performance of the six different models, XGBoost, RNN, BiLSTM, BiGRU, CNN-BiLSTM, and Conv1D-BiLSTM, is compared on the basis of different performance metrics for various stations in China as presented in Tables 4, 5, 6, 7, 8, 9, 10, 11, 12, and 13. The performance metrics measure the deviation of the predicted value from the actual value. The Mean Absolute Error (MAE) and Root Mean Square Error (RMSE) are closer to zero indicating the highest prediction accuracy. The RMSE values obtained from the application of the XGBoost model for Beijing, Chengdu, Chongqing, Dongguan, Guangzhou, Shanghai, Shenyang, Shenzhen, Tianjin, and Wuhan stations are 0.0103, 0.0086, 0.0086, 0.0027, 0.0191, 0.0082, 0.0082, 0.002, 0.0219, and 0,0113. Only for station Tianjin, the RMSE values of XGBoost coincides with that obtained from Conv 1D-BiLSTM, while for all other stations XGBoost has acquired the least RMSE. Therefore, based on the comparison of the evaluation metrics of the various models, it can be concluded that XGBoost outperforms the deep learning models, irrespective of whether it is simple or hybrid.

**Table 4:** Performance evaluation measures of different models for Beijing

Measures/models	Bi-GRU	Bi-LSTM	2-RNN	CNN-BiLSTM	Conv1D-BiLSTM	XGBoost
IA	0.9861	0.9844	0.9840	0.9917	0.9933	0.9989
MAE	0.0273	0.0276	0.0269	0.0202	0.0176	0.0032
MSE	0.0013	0.0015	0.0015	0.0007	0.0006	0.0001
RMSE	0.0361	0.0381	0.0389	0.0272	0.0244	0.0103
SMAPE	0.1219	0.1252	0.1215	0.0919	0.0841	0.0101

**Table 5:** Performance evaluation measures of different models for Chengdu

Measures/models	Bi-GRU	Bi –LSTM	2-RNN	CNN-BiLSTM	Conv1D-BiLSTM	XGBoost
IA	0.9908	0.9917	0.9889	0.9957	0.9976	0.9998
MAE	0.0193	0.0176	0.0211	0.0137	0.0083	0.0006
MSE	0.0007	0.0007	0.0009	0.0003	0.0002	0.00001
RMSE	0.0266	0.0256	0.0292	0.0176	0.0137	0.0033
SMAPE	0.1229	0.1229	0.1316	0.0702	0.0546	0.0047

**Table 6:** Performance evaluation measures of different models for Chongqing

Measures/models	Bi-GRU	Bi-LSTM	2-RNN	CNN-BiLSTM	Conv1D-BiLSTM	XGBoost
IA	0.9910	0.9915	0.9896	0.9951	0.9957	0.9993
MAE	0.0205	0.0215	0.0256	0.0127	0.0107	0.0025
MSE	0.0009	0.0009	0.0010	0.0005	0.0004	0.00007
RMSE	0.0313	0.0299	0.0323	0.0221	0.0212	0.0086
SMAPE	0.1019	0.0999	0.1056	0.0616	0.0555	0.0216

**Table 7:** Performance evaluation measures of different models for Dongguan

Measures/models	Bi-GRU	Bi-LSTM	2-RNN	CNN-BiLSTM	Conv1D-BiLSTM	XGBoost
IA	0.9850	0.9855	0.9834	0.9944	0.9955	0.9999
MAE	0.0237	0.0239	0.0254	0.0143	0.0142	0.0011
MSE	0.0011	0.0009	0.0012	0.0004	0.0003	0.000007
RMSE	0.0327	0.0315	0.0344	0.0191	0.0178	0.0027
SMAPE	0.1021	0.0954	0.1142	0.0477	0.0521	0.0072

**Table 8:** Performance evaluation measures of different models for Guangzhou

Measures/models	Bi-GRU	Bi-LSTM	2-RNN	CNN-BiLSTM	Conv1D-BiLSTM	XGBoost
IA	0.9852	0.9866	0.9688	0.9921	0.9935	0.9953
MAE	0.0235	0.0183	0.0389	0.0164	0.0149	0.0019
MSE	0.0011	0.0009	0.0023	0.0006	0.0005	0.0004
RMSE	0.0332	0.0316	0.0478	0.0254	0.0234	0.0191
SMAPE	0.0781	0.0627	0.1443	0.0539	0.0568	0.0120

**Table 9:** Performance evaluation measures of different models for Shanghai

Measures/models	Bi-GRU	Bi-LSTM	2-RNN	CNN-BiLSTM	Conv1D-BiLSTM	XGBoost
IA	0.9909	0.9903	0.9906	0.9959	0.9969	0.9990
MAE	0.0183	0.0203	0.0182	0.0116	0.0071	0.0025
MSE	0.0007	0.0006	0.0007	0.0003	0.0002	0.00006
RMSE	0.0255	0.0254	0.0258	0.0175	0.0144	0.0082
SMAPE	0.1153	0.1166	0.1232	0.0663	0.0504	0.0202

**Table 10:** Performance evaluation measures of different models for Shenyang

Measures/models	Bi-GRU	Bi-LSTM	2-RNN	CNN-BiLSTM	Conv1D-BiLSTM	XGBoost
IA	0.9878	0.9898	0.9859	0.9903	0.9927	0.9979
MAE	0.0157	0.0127	0.0151	0.0141	0.0127	0.0013
MSE	0.0004	0.0003	0.0005	0.0003	0.0002	0.00006
RMSE	0.0201	0.0184	0.0231	0.0176	0.0151	0.0082
SMAPE	0.1049	0.0962	0.1051	0.0823	0.0722	0.0055

**Table 11:** Performance evaluation measures of different models for Shenzhen

Measures/models	Bi-GRU	Bi-LSTM	2-RNN	CNN-BiLSTM	Conv1D-BiLSTM	XGBoost
IA	0.9808	0.9829	0.9749	0.9896	0.9937	0.9999
MAE	0.0244	0.0228	0.0295	0.0150	0.0134	0.0006
MSE	0.0009	0.0009	0.0012	0.0006	0.0003	0.000004
RMSE	0.0315	0.0299	0.0353	0.0235	0.0175	0.0020
SMAPE	0.0966	0.0887	0.1131	0.0593	0.0549	0.0055

**Table 12:** Performance evaluation measures of different models for Tianjin

Measures/models	Bi-GRU	Bi-LSTM	2-RNN	CNN-BiLSTM	Conv1D-BiLSTM	XGBoost
IA	0.9922	0.9927	0.9773	0.9933	0.9938	0.9928
MAE	0.0168	0.0146	0.0291	0.0134	0.0129	0.0031
MSE	0.0006	0.0006	0.0017	0.0005	0.0005	0.0005
RMSE	0.0249	0.0239	0.0412	0.0227	0.0219	0.0219
SMAPE	0.0748	0.0635	0.1198	0.0592	0.0579	0.0073

**Table 13:** Performance evaluation measures of different models for Wuhan

Measures/models	Bi-GRU	Bi-LSTM	2-RNN	CNN-BiLSTM	Conv1D-BiLSTM	XGBoost
IA	0.9867	0.9873	0.9844	0.9932	0.9944	0.9986
MAE	0.0189	0.0209	0.0273	0.0112	0.0092	0.0024
MSE	0.0012	0.0012	0.0015	0.0006	0.0005	0.0001
RMSE	0.0353	0.0346	0.0385	0.0248	0.0222	0.0113
SMAPE	0.1701	0.1699	0.1895	0.0824	0.0665	0.0114

The overall performance measures for different models are also compared by average rank calculation as shown in Table 14. The XGBoost presents the highest rank in the average rank table (Table 14). Thus, it can be concluded that XGBoost is the best predictive model among the selected models. The algorithmic enhancements and systems optimization are the major reasons for the outstanding performance of the XGBoost model. The XGBoost model’s exceptional performance can be attributed to its ground-breaking algorithmic enhancements and finely tuned systems optimization. The various algorithmic enhancement used by XGBoost includes the presence of regularized gradient boosting, approximate computing, and distributed computing technique. Through meticulous optimization, the XGBoost model has achieved unparalleled precision and accuracy, surpassing all previous benchmarks and raising the bar for machine learning models. The various system optimization technique used in XGBoost is parallelization, approximate algorithms, cache-aware access, block algorithms, out-of-core computation, tree pruning, etc. The innovative algorithmic enhancements have enabled the XGBoost model to learn and adapt with remarkable efficiency, making it a standout model in the field of data science. One of the key advantages of XGBoost over RNNs is its ability to handle high-dimensional and sparse data. XGBoost utilizes an ensemble of decision trees to make predictions, which can handle sparse data more effectively than RNNs. Additionally, XGBoost is computationally efficient, which allows it to train on large datasets in a relatively short amount of time. The decision trees used in XGBoost are easy to interpret and can provide insights into how the model is making predictions. This can be useful in certain industries, such as finance, where understanding the reasoning behind a prediction is crucial. RNNs have a memory component that allows them to retain information from previous time steps, which can be useful in predicting future values in a time series. Additionally, RNNs can handle variable-length sequences, which can be difficult for XGBoost to handle. Ultimately, the choice between XGBoost and RNNs depends on the specific problem at hand and the nature of the data. While XGBoost may be better suited for certain types of data and problems, RNNs may be the better choice for others. It is important to carefully consider the strengths and weaknesses of each algorithm prior to their implementation.

**Table 14:** Average rank based on performance evaluation measures on all selected cities for all models

Measures/models	Bi-GRU	Bi-LSTM	2-RNN	CNN-BiLSTM	Conv1D-BiLSTM	XGBoost
IA	4.8	4.3	5.9	2.9	1.9	1.2
MAE	4.7	4.5	5.4	3	2	1
MSE	4.1	3.8	5	2.8	1.9	1
RMSE	4.8	4	5.9	2.9	1.9	1
SMAPE	4.8	4.3	5.7	2.8	2.2	1

The performance of Conv1D-BiLSTM is the best among the deep-learning models. Next to the XGBoost model, CNN-BiLSTM shows good satisfactory performance for some of the stations. The denoising effect caused by the convolutional layer in the Conv1D-BiLSTM and CNN-BiLSTM models enabled them to perform better than the superior variant of RNN. Hence, the hybrid deep learning models tend to perform better than the simple stand-alone deep learning model.

**Fig. 4:**
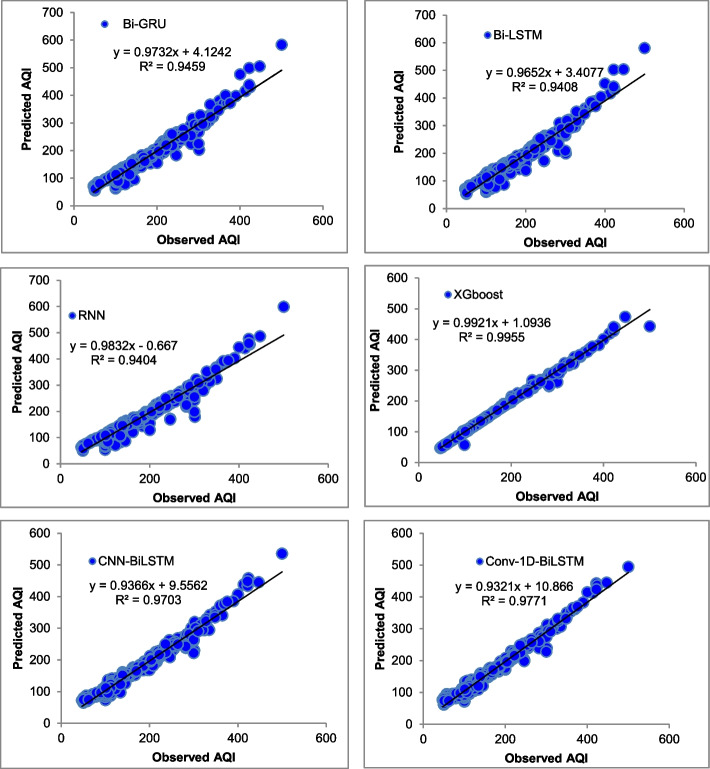
Comparison of observed and predicted AQI values (testing phase) for Beijing

**Fig. 5:**
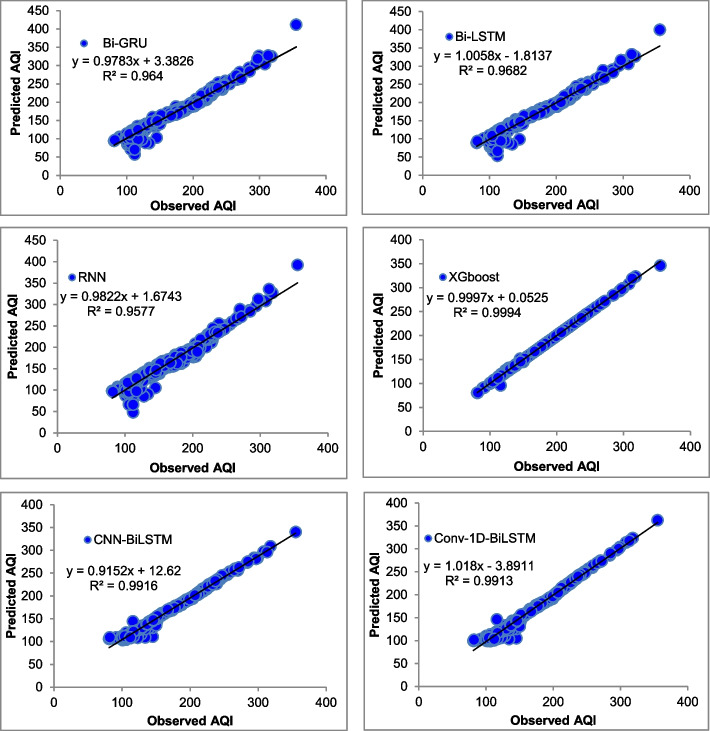
Comparison of observed and predicted AQI values (testing phase) for Chengdu

**Fig. 6:**
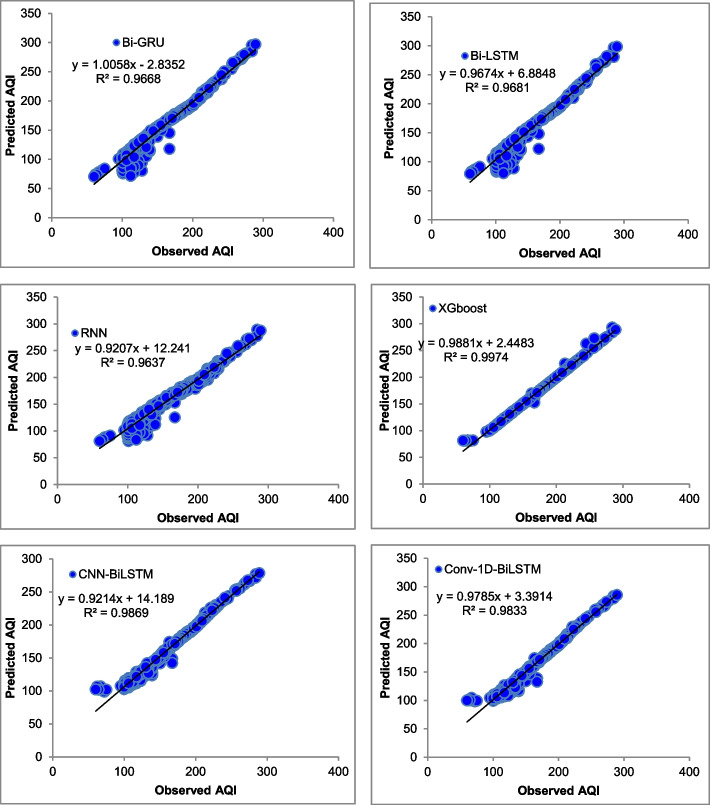
Comparison of observed and predicted AQI values (testing phase) for Chongquin

**Fig. 7:**
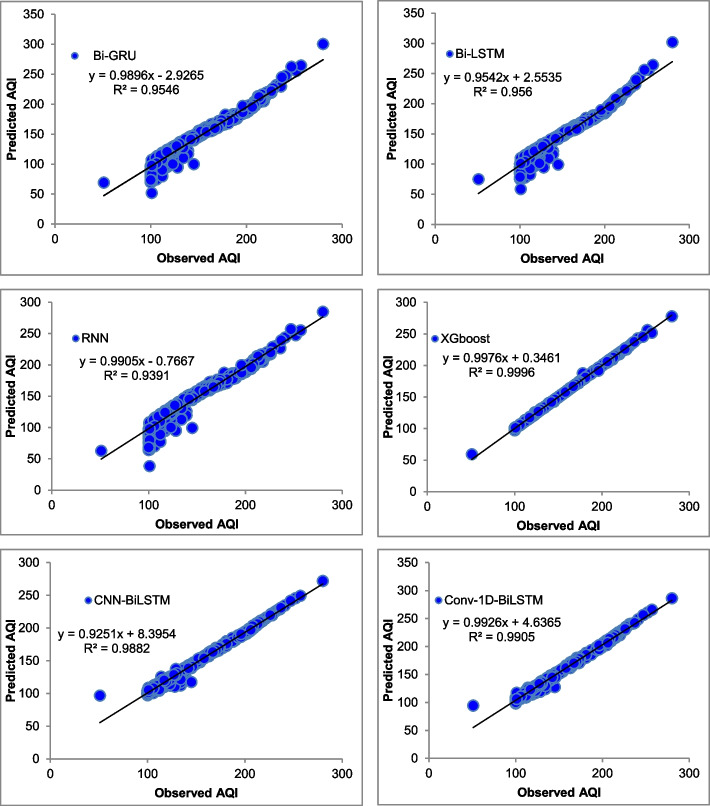
Comparison of observed and predicted AQI values (testing phase) for Dongguan

**Fig. 8:**
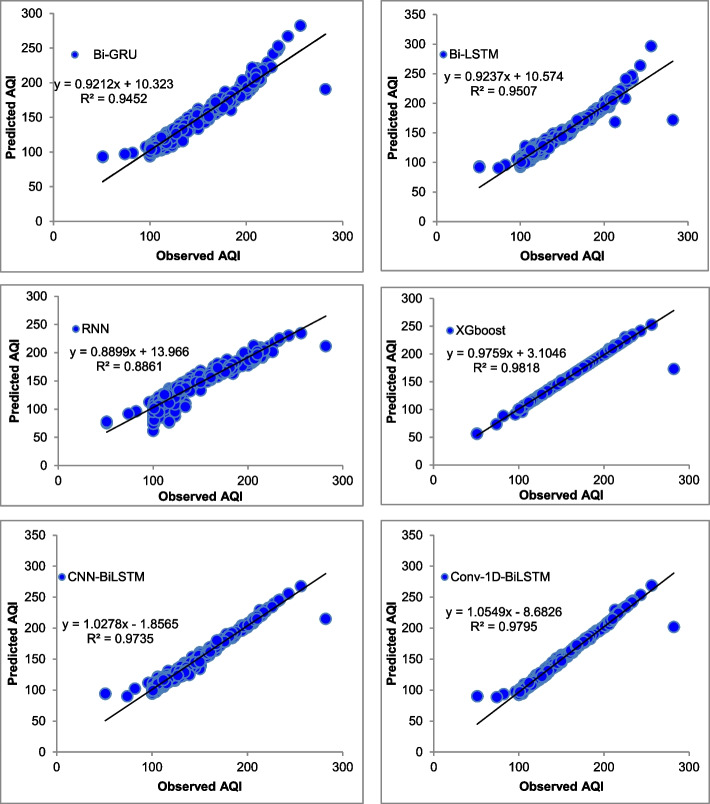
Comparison of observed and predicted AQI values (testing phase) for Guangzhou

**Fig. 9:**
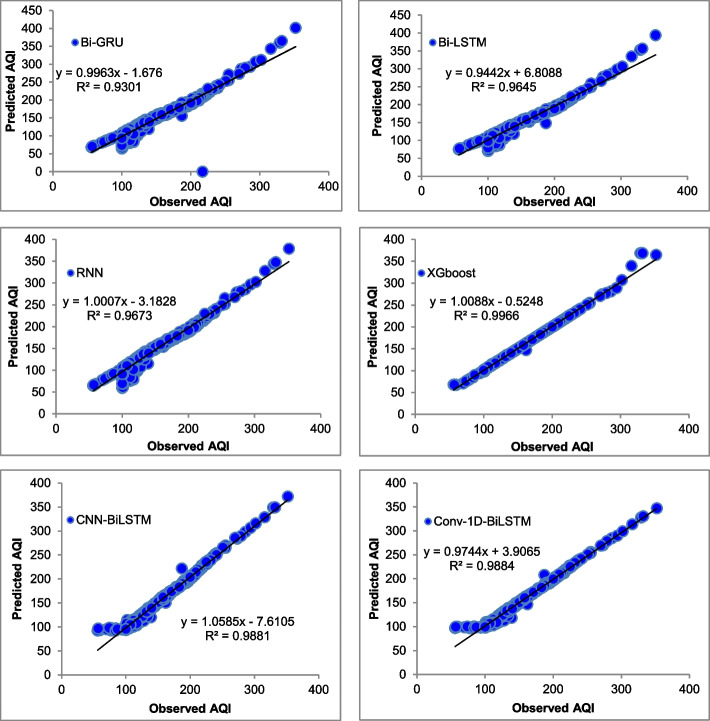
Comparison of observed and predicted AQI values (testing phase) for Shanghai

**Fig. 10:**
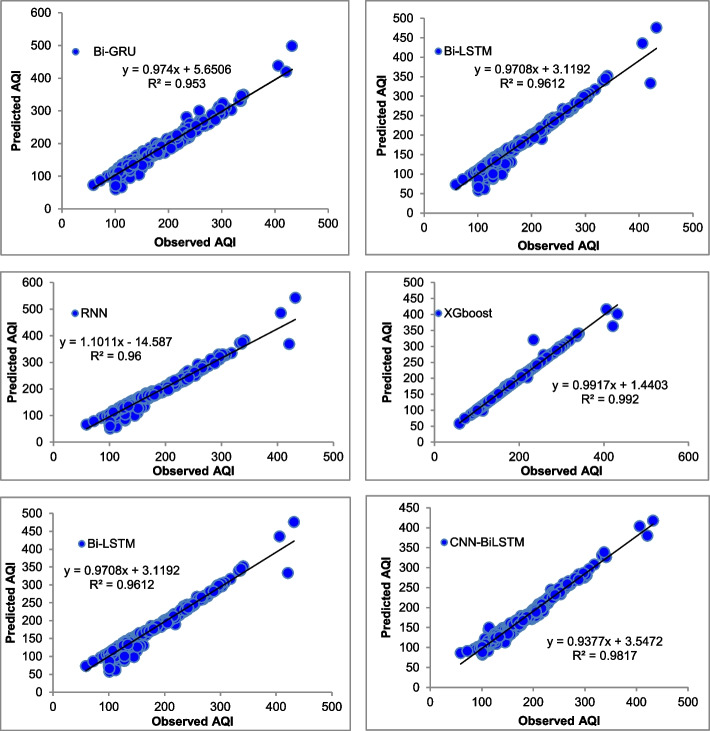
Comparison of observed and predicted AQI values (testing phase) for Shenyang

**Fig. 11:**
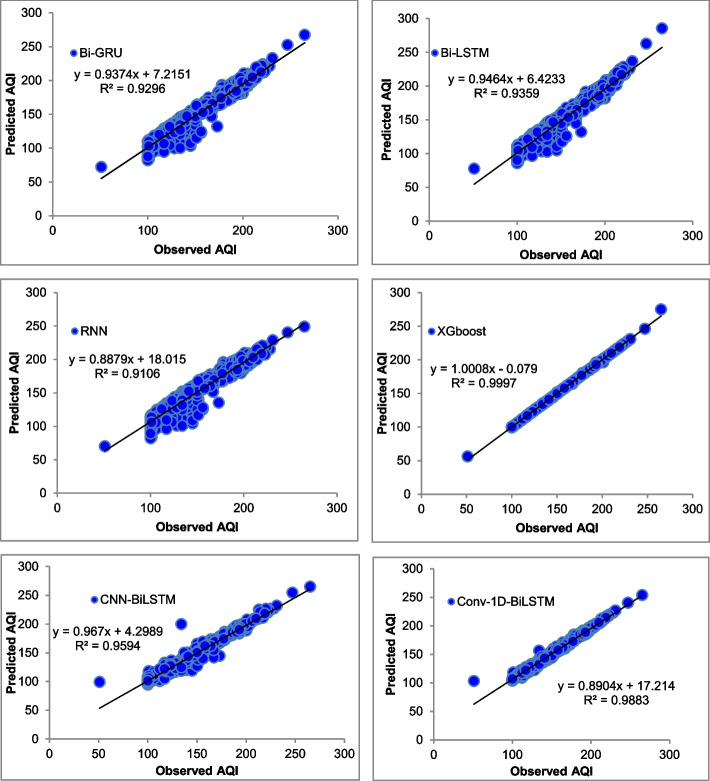
Comparison of observed and predicted AQI values (testing phase) for Shenzhen

**Fig. 12:**
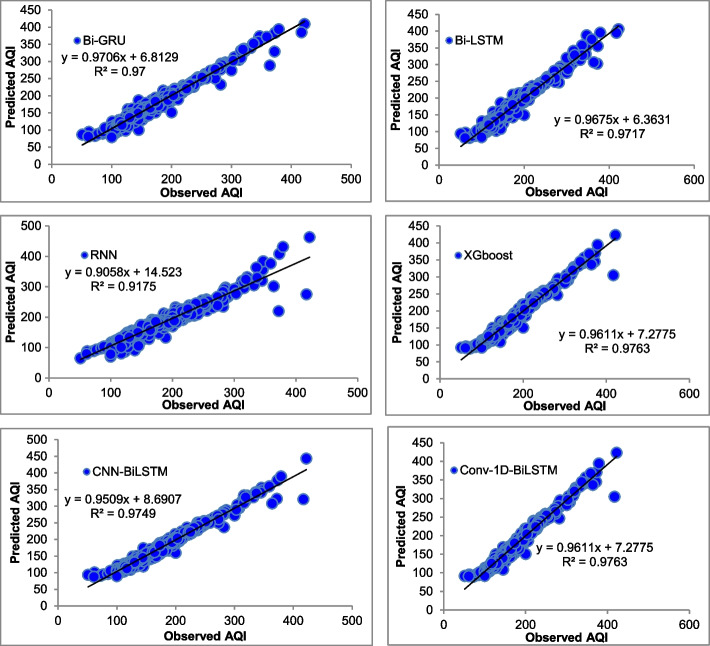
Comparison of observed and predicted AQI values (testing phase) for Tianjin

**Fig. 13:**
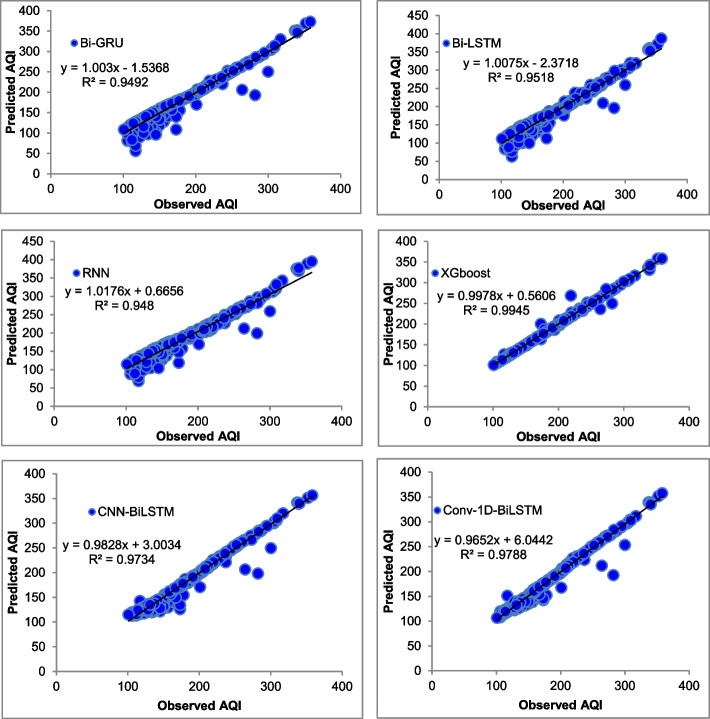
Comparison of observed and predicted AQI values (testing phase) for Wuhan

**Fig. 14:**
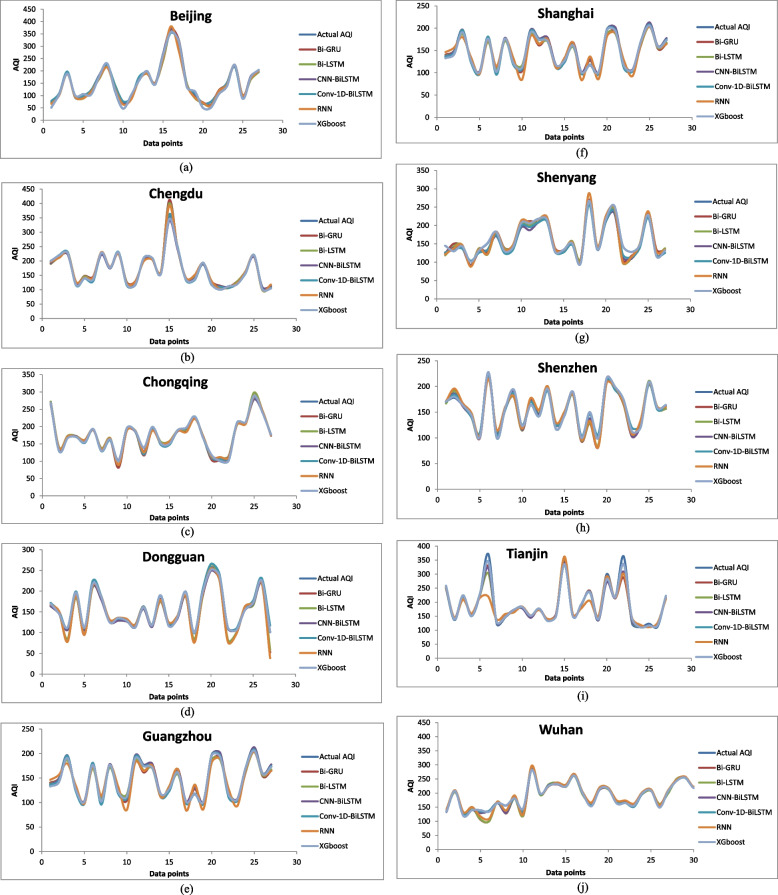
**a–j** Comparison of the performance of the chosen algorithms with the actual AQI values for the various stations

The majority of the models that were chosen fit the dataset and show minor deviations between the predicted and actual AQI values as shown in Figs. 4, 5, 6, 7, 8, 9, 10, 11, 12, and 13. The calculated equation generally minimizes the distance between the fitting line and the data points. The statistical measure *r*-square provides the closeness of data to the fitted regression line, which varies from 0 to 1. The *r*-square values are closer to 1 for most of the models indicating that the model is able to fit the given data. In most of the stations, the *r*-square value of the XGBoost model is closest to 1, therefore, suggesting XGBoost as the better-performing model for all the selected stations. As demonstrated in Fig. 14, the performances of multiple models in predicting AQI levels were evaluated over a specific period of 30 days. Through this analysis, the study sought to identify the most effective models for AQI prediction and to provide insight into the factors influencing air quality levels during the research period. From the comparison plots (Fig. 14 a–j), it is observed that both XGBoost and neural network models are capable of capturing the non-linearity present in the dataset with reliable accuracy. The performance gaps between the different models are quite small with similarity in capturing the generic trend of the air quality index for the ten stations during the testing period. As per the graph, it can be visualized that the XGBoost model has better superimposition among the considered models on the actual AQI value in all selected stations. The RNN and Bi-GRU are the least-performing models among the selected models. Some of the better-performing models include Bi-LSTM, CNN-BiLSTM, and Conv1D-BiLSTM. It is found that the XGBoost is the most significant model to perform efficient AQI forecasting. Hence, the results reveal that the prediction of the air quality index through XGBoost outperforms other deep learning models.

## Conclusion

This study proposes a prediction framework using six different models (deep learning models and XGBoost) to understand the estimation of AQI on the basis of 7 years of data collected from various stations in China. The prediction of AQI was carried out using a machine learning model, namely, XGBoost, and five deep learning models, namely, Bi-GRU, BiLSTM, CNN-BiLSTM, Conv1D-BiLSTM, and RNN. The performance of RNN and Bi-GRU models is poor when compared to the other models. The XGBoost model has outperformed even the deep learning models due to the least RMSE values in the prediction of AQI for the chosen 10 stations. The conv1D-BiLSTM model is found to be the most efficient deep learning model to predict the AQI due to its hybrid nature and performance on par with XGBoost for some stations. This study has analysed the performance of various machine learning, deep learning, and hybrid models which helps us understand the behaviour of these models on the air quality data with a sample size of 7 years. These hybrid models combine the best characteristic of the base model. The performance of hybrid models such as Conv1D-BiLSTM and CNN-BiLSTM is better than that of the base models for some stations. While in other stations, XGBoost outperforms other considered models due to the consideration of a smaller sample size. Therefore, such an analytical study will help us understand the merits of the considered models under certain circumstances. Furthermore, this study provides insight into the effectiveness of different predictive models for air quality, which can be useful for policymakers and city planners to design effective strategies to mitigate air pollution. The results also show that XGBoost is preferable to deep learning models for the prediction of air quality in several Chinese cities. This study also demonstrates the promise of deep learning models for effectively modelling data’s inherent non-linearities and boosting the precision of the prediction. The findings augment knowledge in the field of environmental engineering as it illustrates the application of machine learning and deep learning techniques in this field. Hence, the prediction of AQI using machine learning and deep learning models can enable environmentalists to take appropriate actions to minimize air pollution in the cities. The study also carries a few limitations. An efficient prediction of the air quality index is not possible by analysing limited pollutants such as SO2, CO, NO2, O3, PM2.5, and PM10. Consistent availability of data is very important in obtaining an efficient predictive model but the unavailability of consistent environmental data is one of the huddles of such air pollution prediction studies.

## Abbreviations

ANN: Artificial Neural Network
AQI: Air Quality Index
ANFIS: Adaptive Neuro Fuzzy Inference System
Bi-GRU: Bidirectional Gated Recurrent unit
BiLSTM: Bidirectional Long Short-Term Memory
BRT: Boosted Regression Trees
COPD: Chronic obstructive pulmonary disease
CNN: Convolutional Neural Network
Conv1D-BiLSTM: Convolutional BiLSTM
CO: Carbon monoxide
DL: Deep Learning
DRNN: Deep Recurrent Neural Network
GBDT: Gradient Boost Decision Trees
GPR: Guassian Process Regression
IA: Index of Agreement
L-SVM: Linear-Support Vector Machine
LSTM: Long Short-Term Memory
M-SVM: Medium Gaussian-Support Vector Machine
MAE: Maximum Absolute Error
MSE: Mean Square Error
NO_2_: Nitrogen dioxide
NO_X_: Oxides of nitrogen
O_3_: Ozone
OLS: Ordinary Least Squares
PSO: Particle Swarm Optimisation
PM_2.5_: Particulate matter with 2.5 microns
PM_10_: Particulate matter with 10 microns
QR: Quantile regression
RMSE: Root Mean Square Error
RFR: Random Forest Regression
RNN: Recurrent Neural Network
SO_2_: Sulphur dioxide
SVM: Support Vector Machine
STDL: Spatio-Temporal Deep Learning
STANN: Spatio-Temporal Artificial Neural Network
STSVR: Spatio-Temporal Support Vector Regression
STARMA: Spatio-Temporal Autoregressive Moving Average
SMAPE: Symmetric Mean Absolute Percentage Error
XGBoost: EXtreme Gradient Boosting
